# Panelist

**DOI:** 10.1002/alz70856_106089

**Published:** 2026-01-08

**Authors:** Jeffrey L. Cummings

**Affiliations:** ^1^ Kirk Kerkorian School of Medicine, University of Nevada Las Vegas, Las Vegas, NV, USA

## Abstract

Dr. Cummings will be a panelist.

